# 3-Hydroxy­adamantane-1-acetic acid

**DOI:** 10.1107/S1600536808020515

**Published:** 2008-08-06

**Authors:** Xiao-Hong Geng, Li-Chun Kong, Yun-Long Feng

**Affiliations:** a Zhejiang Key Laboratory for Reactive Chemistry on Solid Surfaces, Institute of Physical Chemistry, Zhejiang Normal University, Jinhua, Zhejiang 321004, People’s Republic of China

## Abstract

The crystal structure of the title adamantane derivative, C_12_H_18_O_3_, has been determined by X-ray diffraction. The structure is stabilized by inter­molecular O—H⋯O hydrogen bonds, forming a chain.

## Related literature

For related literature, see: Lu & Yang (1996 ▶); Tukada & Mochizuki (2005 ▶); Zhao *et al.* (2003 ▶).
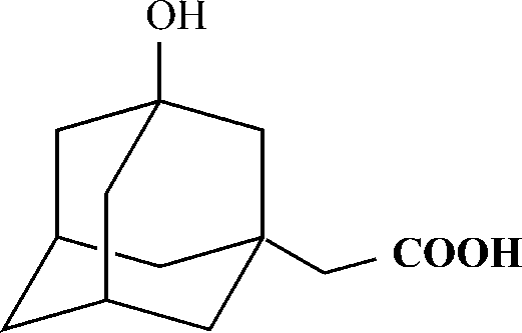


## Experimental

### 

#### Crystal data

C_12_H_18_O_3_
*M* *_r_* = 210.26
Triclinic, 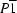

*a* = 6.5120 (9) Å
*b* = 7.9485 (11) Å
*c* = 11.5469 (15) Å
α = 106.919 (10)°
β = 94.838 (10)°
γ = 104.443 (7)°
*V* = 545.73 (13) Å^3^
*Z* = 2
Mo *K*α radiation
μ = 0.09 mm^−1^
*T* = 296 (2) K
0.30 × 0.13 × 0.10 mm

#### Data collection

Bruker APEXII area-detector diffractometer
Absorption correction: multi-scan (*SADABS*; Sheldrick, 1996 ▶) *T*_min_ = 0.98, *T*_max_ = 0.99
8786 measured reflections
2488 independent reflections
1574 reflections with *I* > 2σ(*I*)
*R*_int_ = 0.033

#### Refinement

*R*[*F* ^2^ > 2σ(*F* ^2^)] = 0.048
*wR*(*F* ^2^) = 0.130
*S* = 1.03
2488 reflections
142 parameters
2 restraints
H atoms treated by a mixture of independent and constrained refinement
Δρ_max_ = 0.18 e Å^−3^
Δρ_min_ = −0.17 e Å^−3^

### 

Data collection: *APEX2* (Bruker, 2006 ▶); cell refinement: *SAINT* (Bruker, 2006 ▶); data reduction: *SAINT*; program(s) used to solve structure: *SHELXS97* (Sheldrick, 2008 ▶); program(s) used to refine structure: *SHELXL97* (Sheldrick, 2008 ▶); molecular graphics: *SHELXTL* (Sheldrick, 2008 ▶); software used to prepare material for publication: *SHELXTL*.

## Supplementary Material

Crystal structure: contains datablocks I, global. DOI: 10.1107/S1600536808020515/at2572sup1.cif

Structure factors: contains datablocks I. DOI: 10.1107/S1600536808020515/at2572Isup2.hkl

Additional supplementary materials:  crystallographic information; 3D view; checkCIF report

## Figures and Tables

**Table 1:** Hydrogen-bond geometry (Å, °)

*D*—H⋯*A*	*D*—H	H⋯*A*	*D*⋯*A*	*D*—H⋯*A*
O1—H1⋯O3^i^	0.815 (15)	1.995 (16)	2.7959 (18)	168 (2)
O2—H2⋯O1^ii^	0.850 (16)	1.811 (16)	2.649 (2)	168 (2)

Symmetry codes: (i) 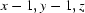; (ii) 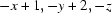.

**Fig. 1.**
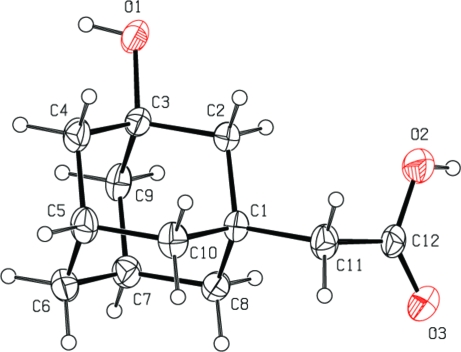
A view of the molecule of (I), showing the atom-labelling scheme. Displacement ellipsoids are shown at the 30% probability level.

**Fig. 2.**
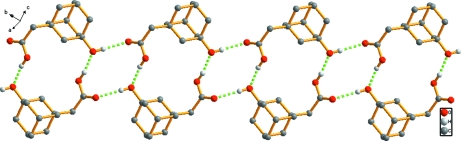
A packing diagram for the title compound. The O—H···O interactions are depicted by dashed lines.

## supplementary crystallographic information

### Comment

Adamantane and its derivatives have an extensive application in the field of medicine. For instance, adamantaneamine has an obvious effect on controlling the exuviating the influenza A virus and it can alleviate the Parkinson symptom (Lu *et al.*, 1996). A large number of compounds containing amantadine have been synthesized (Tukada & Mochizuki, 2005; Zhao *et al.*, 2003). Here we report the synthesis and crystal structure of the title compound (I), illustrated in Fig. 1.

The title compound (I) is an adamantane derivative. Single-crystal X-ray diffraction analyses demonstrate that hydrogen bonding produces an extensive polymeric network since the hydroxyl group substituents are simultaneously hydrogen bonded to the OH of the carboxyl group on an adjacent molecule and the carbonyl group of a different neighbor forming a 12-membered ring as shown in Fig. 2. A 16-membered ring is formed by intermolecular hydrogen bonding between the carbonyl O and hydroxyl H atoms of two molecules. The alternating 12- and 16-membered rings make the compound to form a one-dimensional network.

### Experimental

The 3-hydroxy-1-adamantaneacetic was obtained from Zhejiang Key Laboratory for Reactive Chemistry on Solid Surface. Crystals suitable for single-crystal X-ray diffraction were grown by slow evaporation of distilled water.

### Refinement

The H atoms bonded to C atoms were positioned geometrically [C—H = 0.97 Å, *U*_iso_(H) = 1.2*U*_eq_(C)]. The H atoms bonded to O atoms were located in a difference Fourier maps and refined with O—H distance restraints of 0.82 and *U*_iso_(H) = 1.2*U*_eq_(O).

### Crystal data

**Table:**

C_12_H_18_O_3_	*Z* = 2
*M**_r_* = 210.26	*F*_000_ = 228
Triclinic, *P*1	*D*_x_ = 1.280 Mg m^−^^3^
Hall symbol: -P 1	Mo *K*α radiation λ = 0.71073 Å
*a* = 6.5120 (9) Å	Cell parameters from 1598 reflections
*b* = 7.9485 (11) Å	θ = 1.9–27.5º
*c* = 11.5469 (15) Å	µ = 0.09 mm^−^^1^
α = 106.919 (10)º	*T* = 296 (2) K
β = 94.838 (10)º	Block, colourless
γ = 104.443 (7)º	0.30 × 0.13 × 0.10 mm
*V* = 545.73 (13) Å^3^	

### Data collection

**Table:**

Bruker APEXII area-detector diffractometer	2488 independent reflections
Radiation source: fine-focus sealed tube	1574 reflections with *I* > 2σ(*I*)
Monochromator: graphite	*R*_int_ = 0.033
*T* = 296(2) K	θ_max_ = 27.5º
ω scans	θ_min_ = 1.9º
Absorption correction: multi-scan(SADABS; Sheldrick, 1996)	*h* = −8→8
*T*_min_ = 0.98, *T*_max_ = 0.99	*k* = −10→10
8786 measured reflections	*l* = −15→15

### Refinement

**Table:**

Refinement on *F*^2^	Primary atom site location: structure-invariant direct methods
Least-squares matrix: full	Secondary atom site location: difference Fourier map
*R*[*F*^2^ > 2σ(*F*^2^)] = 0.048	Hydrogen site location: inferred from neighbouring sites
*wR*(*F*^2^) = 0.130	H atoms treated by a mixture of independent and constrained refinement
*S* = 1.03	*w* = 1/[σ^2^(*F*_o_^2^) + (0.052*P*)^2^ + 0.1083*P*] where *P* = (*F*_o_^2^ + 2*F*_c_^2^)/3
2488 reflections	(Δ/σ)_max_ < 0.001
142 parameters	Δρ_max_ = 0.18 e Å^−^^3^
2 restraints	Δρ_min_ = −0.17 e Å^−^^3^

### Special details

**Table:**

Geometry. All e.s.d.'s (except the e.s.d. in the dihedral angle between two l.s. planes) are estimated using the full covariance matrix. The cell e.s.d.'s are taken into account individually in the estimation of e.s.d.'s in distances, angles and torsion angles; correlations between e.s.d.'s in cell parameters are only used when they are defined by crystal symmetry. An approximate (isotropic) treatment of cell e.s.d.'s is used for estimating e.s.d.'s involving l.s. planes.
Refinement. Refinement of *F*^2^ against ALL reflections. The weighted *R*-factor *wR* and goodness of fit *S* are based on *F*^2^, conventional *R*-factors *R* are based on *F*, with *F* set to zero for negative *F*^2^. The threshold expression of *F*^2^ > σ(*F*^2^) is used only for calculating *R*-factors(gt) *etc*. and is not relevant to the choice of reflections for refinement. *R*-factors based on *F*^2^ are statistically about twice as large as those based on *F*, and *R*- factors based on ALL data will be even larger.

### Fractional atomic coordinates and isotropic or equivalent isotropic displacement parameters (Å^2^)

**Table:**

	*x*	*y*	*z*	*U*_iso_*/*U*_eq_	
O1	0.0947 (2)	0.76522 (17)	0.09607 (12)	0.0581 (4)	
H1	0.024 (3)	0.690 (3)	0.1232 (19)	0.070*	
O2	0.5777 (2)	1.30404 (19)	0.01092 (13)	0.0620 (4)	
H2	0.694 (3)	1.295 (3)	−0.015 (2)	0.074*	
O3	0.7982 (2)	1.51594 (19)	0.17220 (13)	0.0647 (4)	
C4	−0.0106 (3)	0.9805 (3)	0.26199 (16)	0.0462 (4)	
H4B	−0.0679	0.8858	0.2976	0.055*	
H4A	−0.1258	0.9836	0.2043	0.055*	
C9	0.3516 (3)	0.9321 (2)	0.28586 (16)	0.0444 (4)	
H9B	0.4665	0.9027	0.2431	0.053*	
H9A	0.2979	0.8377	0.3223	0.053*	
C3	0.1714 (3)	0.9376 (2)	0.19572 (15)	0.0392 (4)	
C2	0.2580 (3)	1.0849 (2)	0.13783 (15)	0.0387 (4)	
H2B	0.1441	1.0871	0.0790	0.046*	
H2A	0.3724	1.0562	0.0943	0.046*	
C8	0.5237 (3)	1.2664 (2)	0.32771 (15)	0.0413 (4)	
H8B	0.5804	1.3850	0.3913	0.050*	
H8A	0.6399	1.2391	0.2854	0.050*	
C7	0.4377 (3)	1.1188 (2)	0.38625 (16)	0.0441 (4)	
H7A	0.5541	1.1154	0.4444	0.053*	
C6	0.2554 (3)	1.1612 (3)	0.45363 (16)	0.0494 (5)	
H6B	0.2012	1.0676	0.4906	0.059*	
H6A	0.3086	1.2787	0.5185	0.059*	
C5	0.0756 (3)	1.1668 (2)	0.36319 (16)	0.0458 (5)	
H5A	−0.0411	1.1949	0.4065	0.055*	
C10	0.1616 (3)	1.3152 (2)	0.30564 (17)	0.0464 (5)	
H10B	0.2146	1.4338	0.3694	0.056*	
H10A	0.0462	1.3203	0.2491	0.056*	
C1	0.3444 (3)	1.2738 (2)	0.23674 (14)	0.0352 (4)	
C11	0.4224 (3)	1.4276 (2)	0.18016 (16)	0.0457 (5)	
H11A	0.3065	1.4201	0.1186	0.055*	
H11B	0.4522	1.5452	0.2441	0.055*	
C12	0.6181 (3)	1.4222 (2)	0.12218 (16)	0.0436 (4)	

### Atomic displacement parameters (Å^2^)

**Table:**

	*U*^11^	*U*^22^	*U*^33^	*U*^12^	*U*^13^	*U*^23^
O1	0.0652 (10)	0.0397 (7)	0.0542 (8)	−0.0080 (6)	0.0268 (7)	0.0076 (6)
O2	0.0614 (10)	0.0572 (9)	0.0552 (9)	0.0018 (7)	0.0240 (7)	0.0091 (7)
O3	0.0584 (10)	0.0567 (9)	0.0637 (9)	−0.0088 (7)	0.0151 (7)	0.0171 (7)
C4	0.0361 (10)	0.0536 (11)	0.0504 (11)	0.0072 (8)	0.0157 (8)	0.0217 (9)
C9	0.0466 (11)	0.0412 (10)	0.0567 (11)	0.0145 (8)	0.0201 (9)	0.0279 (8)
C3	0.0391 (10)	0.0350 (9)	0.0390 (9)	0.0028 (7)	0.0129 (7)	0.0099 (7)
C2	0.0364 (10)	0.0412 (9)	0.0369 (9)	0.0050 (7)	0.0088 (7)	0.0147 (7)
C8	0.0393 (10)	0.0392 (9)	0.0429 (9)	0.0043 (8)	0.0046 (8)	0.0162 (8)
C7	0.0427 (11)	0.0492 (10)	0.0432 (10)	0.0095 (8)	0.0026 (8)	0.0237 (8)
C6	0.0646 (13)	0.0448 (10)	0.0387 (10)	0.0092 (9)	0.0153 (9)	0.0171 (8)
C5	0.0464 (11)	0.0484 (10)	0.0510 (11)	0.0180 (9)	0.0251 (9)	0.0202 (9)
C10	0.0498 (11)	0.0471 (10)	0.0519 (11)	0.0210 (9)	0.0181 (9)	0.0218 (9)
C1	0.0360 (9)	0.0351 (9)	0.0392 (9)	0.0098 (7)	0.0110 (7)	0.0179 (7)
C11	0.0564 (12)	0.0384 (10)	0.0507 (10)	0.0156 (9)	0.0175 (9)	0.0228 (8)
C12	0.0575 (13)	0.0317 (9)	0.0449 (10)	0.0066 (9)	0.0153 (9)	0.0208 (8)

### Geometric parameters (Å, °)

**Table:**

O1—C3	1.447 (2)	C8—C7	1.532 (2)
O1—H1	0.815 (15)	C8—H8B	0.9700
O2—C12	1.311 (2)	C8—H8A	0.9700
O2—H2	0.850 (16)	C7—C6	1.527 (3)
O3—C12	1.210 (2)	C7—H7A	0.9800
C4—C3	1.519 (2)	C6—C5	1.520 (3)
C4—C5	1.530 (2)	C6—H6B	0.9700
C4—H4B	0.9700	C6—H6A	0.9700
C4—H4A	0.9700	C5—C10	1.529 (2)
C9—C3	1.519 (2)	C5—H5A	0.9800
C9—C7	1.528 (2)	C10—C1	1.536 (2)
C9—H9B	0.9700	C10—H10B	0.9700
C9—H9A	0.9700	C10—H10A	0.9700
C3—C2	1.525 (2)	C1—C11	1.546 (2)
C2—C1	1.532 (2)	C11—C12	1.493 (2)
C2—H2B	0.9700	C11—H11A	0.9700
C2—H2A	0.9700	C11—H11B	0.9700
C8—C1	1.528 (2)		
			
C3—O1—H1	107.3 (15)	C9—C7—H7A	109.5
C12—O2—H2	110.6 (16)	C8—C7—H7A	109.5
C3—C4—C5	109.05 (14)	C5—C6—C7	109.37 (13)
C3—C4—H4B	109.9	C5—C6—H6B	109.8
C5—C4—H4B	109.9	C7—C6—H6B	109.8
C3—C4—H4A	109.9	C5—C6—H6A	109.8
C5—C4—H4A	109.9	C7—C6—H6A	109.8
H4B—C4—H4A	108.3	H6B—C6—H6A	108.2
C3—C9—C7	109.47 (13)	C6—C5—C10	109.57 (15)
C3—C9—H9B	109.8	C6—C5—C4	109.75 (14)
C7—C9—H9B	109.8	C10—C5—C4	109.33 (14)
C3—C9—H9A	109.8	C6—C5—H5A	109.4
C7—C9—H9A	109.8	C10—C5—H5A	109.4
H9B—C9—H9A	108.2	C4—C5—H5A	109.4
O1—C3—C9	110.50 (14)	C5—C10—C1	110.38 (13)
O1—C3—C4	110.82 (13)	C5—C10—H10B	109.6
C9—C3—C4	109.93 (14)	C1—C10—H10B	109.6
O1—C3—C2	106.48 (13)	C5—C10—H10A	109.6
C9—C3—C2	109.35 (13)	C1—C10—H10A	109.6
C4—C3—C2	109.69 (14)	H10B—C10—H10A	108.1
C3—C2—C1	110.46 (12)	C8—C1—C2	108.35 (13)
C3—C2—H2B	109.6	C8—C1—C10	108.54 (13)
C1—C2—H2B	109.6	C2—C1—C10	108.65 (14)
C3—C2—H2A	109.6	C8—C1—C11	111.84 (14)
C1—C2—H2A	109.6	C2—C1—C11	111.65 (13)
H2B—C2—H2A	108.1	C10—C1—C11	107.71 (13)
C1—C8—C7	110.32 (13)	C12—C11—C1	115.10 (13)
C1—C8—H8B	109.6	C12—C11—H11A	108.5
C7—C8—H8B	109.6	C1—C11—H11A	108.5
C1—C8—H8A	109.6	C12—C11—H11B	108.5
C7—C8—H8A	109.6	C1—C11—H11B	108.5
H8B—C8—H8A	108.1	H11A—C11—H11B	107.5
C6—C7—C9	108.98 (14)	O3—C12—O2	122.43 (17)
C6—C7—C8	109.93 (14)	O3—C12—C11	123.93 (17)
C9—C7—C8	109.30 (13)	O2—C12—C11	113.63 (17)
C6—C7—H7A	109.5		
			
C7—C9—C3—O1	176.98 (12)	C3—C4—C5—C10	60.50 (18)
C7—C9—C3—C4	−60.38 (17)	C6—C5—C10—C1	60.28 (19)
C7—C9—C3—C2	60.09 (17)	C4—C5—C10—C1	−60.04 (19)
C5—C4—C3—O1	−177.88 (13)	C7—C8—C1—C2	−59.16 (17)
C5—C4—C3—C9	59.68 (17)	C7—C8—C1—C10	58.66 (17)
C5—C4—C3—C2	−60.59 (18)	C7—C8—C1—C11	177.35 (14)
O1—C3—C2—C1	−179.80 (13)	C3—C2—C1—C8	59.39 (17)
C9—C3—C2—C1	−60.39 (17)	C3—C2—C1—C10	−58.36 (17)
C4—C3—C2—C1	60.23 (18)	C3—C2—C1—C11	−177.01 (14)
C3—C9—C7—C6	60.25 (16)	C5—C10—C1—C8	−59.19 (18)
C3—C9—C7—C8	−59.91 (18)	C5—C10—C1—C2	58.44 (18)
C1—C8—C7—C6	−59.55 (18)	C5—C10—C1—C11	179.54 (14)
C1—C8—C7—C9	60.02 (18)	C8—C1—C11—C12	53.8 (2)
C9—C7—C6—C5	−60.32 (17)	C2—C1—C11—C12	−67.8 (2)
C8—C7—C6—C5	59.45 (18)	C10—C1—C11—C12	172.94 (15)
C7—C6—C5—C10	−59.72 (18)	C1—C11—C12—O3	−98.6 (2)
C7—C6—C5—C4	60.34 (18)	C1—C11—C12—O2	80.82 (19)
C3—C4—C5—C6	−59.71 (17)		

### Hydrogen-bond geometry (Å, °)

**Table:**

*D*—H···*A*	*D*—H	H···*A*	*D*···*A*	*D*—H···*A*
O1—H1···O3^i^	0.815 (15)	1.995 (16)	2.7959 (18)	168 (2)
O2—H2···O1^ii^	0.850 (16)	1.811 (16)	2.649 (2)	168 (2)

Symmetry codes: (i) *x*−1, *y*−1, *z*; (ii) −*x*+1, −*y*+2, −*z*.
